# Metformin Hydrochloride-Loaded PLGA Nanoparticle in Periodontal Disease Experimental Model Using Diabetic Rats

**DOI:** 10.3390/ijms19113488

**Published:** 2018-11-06

**Authors:** Aline de Sousa Barbosa Freitas Pereira, Gerly Anne de Castro Brito, Maria Laura de Souza Lima, Arnóbio Antônio da Silva Júnior, Emanuell dos Santos Silva, Adriana Augusto de Rezende, Raul Hernandes Bortolin, Maria Galvan, Flávia Q. Pirih, Raimundo Fernandes de Araújo Júnior, Caroline Addison Carvalho Xavier de Medeiros, Gerlane Coelho Bernando Guerra, Aurigena Antunes de Araújo

**Affiliations:** 1 Post-Graduation Program in Oral Science, Department of Dentistry, UFRN, Natal 59072-970, Brazil; aly_line@hotmail.com (A.d.S.B.F.P.); mlauradesouzalima@gmail.com (M.L.d.S.L.); 2 Post-Graduation Program in Pharmacology/Post-Graduation Program in Morphology, Department of Morphology, UFC, Fortaleza 60440-900, Brazil; gerlybrito@hotmail.com; 3 Post-Graduation Program in Pharmaceutical Science/Post-Graduation Program in Health Science, Department of Pharmacology, UFRN, Natal 59072-970, Brazil; arnobiosilva@gmail.com; 4 Post-Graduation Program in Pharmaceutical Science, UFRN, Department of Pharmacology, Natal 59072-970, Brazil; emanuell_111@hotmail.com (E.d.S.S.); adrirezende@yahoo.com (A.A.d.R.); raulhbortolin@yahoo.com.br (R.H.B.); 5 Periodontics Section, School of Dentistry, University of California, UCLA, Los Angeles, CA 90095, USA; mgalvan@dentistry.ucla.edu (M.G.); fpirih@dentistry.ucla.edu (F.Q.P.); 6 Post-Graduation Program in Functional and Structural Biology/Post-Graduation Program Health Science, Department of Morphology, UFRN, Natal 59072-970, Brazil; araujojra@cb.ufrn.br; 7 Post-Graduation Program Biological Science/Post-Graduation Program in RENORBIO, Department of Biophysics and Pharmacology, UFRN, Natal 59072-970, Brazil; carolineaddisonfarma@yahoo.com.br; 8 Post-Graduation Program Biological Science/Post-Graduation Program in Pharmaceutical Science, Department of Biophysics and Pharmacology, UFRN, Natal 59072-970, Brazil; gerlaneguerra@hotmail.com; 9 Post-Graduation Program Oral Science/Post-Graduation Program in Pharmaceutical Science, Department of Biophysics and Pharmacology, UFRN, Natal 59072-970, Brazil; 10 Departamento de Biofisica e Farmacologia, Av. Senador Salgado Filho, S/N, Campus Universitário, Lagoa Nova, UFRN, Natal 59072-970, Brazil

**Keywords:** nanoparticles, poly lactic-*co*-glycolic acid, metformin, periodontal disease, inflammation

## Abstract

Evidence shows that metformin is an antidiabetic drug, which can exert favorable anti-inflammatory effects and decreased bone loss. The development of nanoparticles for metformin might be useful for increased therapeutic efficacy. The aim of this study was to evaluate the effect of metformin hydrochloride-loaded Poly (d,l-Lactide-*co*-glycolide) (PLGA)/(MET-loaded PLGA) on a ligature-induced periodontitis model in diabetic rats. MET-loaded PLGA were characterized by mean diameter, particle size, polydispensity index, and entrapment efficiency. Maxillae were scanned using Microcomputed Tomography (µCT) and histopathological and immunohistochemical analysis. IL-1β and TNF-α levels were analyzed by ELISA immunoassay. Quantitative RT-PCR was used (*AMPK*, *NF-κB p65*, *HMGB1*, and *TAK-1*). The mean diameter of MET-loaded PLGA nanoparticles was in a range of 457.1 ± 48.9 nm (*p* < 0.05) with a polydispersity index of 0.285 (*p* < 0.05), Z potential of 8.16 ± 1.1 mV (*p* < 0.01), and entrapment efficiency (EE) of 66.7 ± 3.73. Treatment with MET-loaded PLGA 10 mg/kg showed low inflammatory cells, weak staining by RANKL, cathepsin K, OPG, and osteocalcin, and levels of IL-1β and TNF-α (*p* < 0.05), increased *AMPK* expression gene (*p* < 0.05) and decreased *NF-κB p65*, *HMGB1*, and *TAK-1* (*p* < 0.05). It is concluded that MET-loaded PLGA decreased inflammation and bone loss in periodontitis in diabetic rats.

## 1. Introduction

Polymeric nanoparticles are particles with a diameter between 1 and 1000 nm [1]. In recent years, nanoparticulate drug release systems using biodegradable polymers have been extensively studied for various applications [2,3]. Nanoparticles may also offer advantages, such as increased therapeutic efficacy, prolonged and controlled release of the drug, decreased toxicity, as well as stability and lower drug decomposition [4,5].

Among the polymers studied for nanoparticle preparation, poly lactic-*co*-glycolic acid (PLGA) has been widely used because it is a biocompatible and biodegradable synthetic polymer that has been approved by the United States Food and Drug Administration (USFDA) [6].

Polymer composition is the most important factor to determine the hydrophilicity and degradation rate of a delivery matrix. The amount of glycolic acid is a critical parameter in tuning the hydrophilicity of the matrix and therefore the degradation and drug release rate [7].

Biguanides are an important class of oral hypoglycemic agents and act by inhibiting gluconeogenesis in the liver, increasing the density of low and high affinity receptors for insulin, and decreasing resistance to the peripheral effects of insulin [8]. Nanoparticles have been investigated as the delivery systems for a wide number of drugs. They have the advantages of high stability in lyophilized or appropriate formulation, feasibility for incorporating both hydrophobic and hydrophilic active substances, high carrier capacity of many drug molecules, and feasibility for various administration routes [9]. Metformin is the most widely prescribed oral antihyperglycemic agent for the treatment of type 2 diabetes [10], as it has a slow and incomplete absorption following oral administration. The development of drug delivery system (e.g., nanoparticles) strategies for metformin might be useful to reduce the doses [11].

The treatment in diabetes patients with metformin has shown that there is reduced TNF-α expression [12], with confirmed anti-inflammatory activity [13]. In periodontal research, animal models should enable the validation of hypotheses and prove the safety and efficacy of new regenerating approaches using biomaterials, growth factors, or stem cells. Experimental animal models are critical tools to investigate mechanisms of periodontal pathogenesis and test new therapeutic approaches. The ligature-induced periodontitis model has been used frequently in relatively large animals, including rodents, to assess the host response and its effects on the tooth-supporting tissues (gingiva and bone) under well-controlled conditions. The use of a ligature-induced periodontitis model is reliable and reproducible over a period of 10 days in rodents [14]. The effect of metformin on a periodontal disease experimental model was previously confirmed by our group [15]. However, it is important to consider that the animals in this study were not diabetic, since our objective was to verify the pleiotropic effect of metformin in inflammation and bone loss in a periodontal disease experimental model. Based on these previous findings from our group, our hypothesis is that when metformin is used in the periodontal disease model in diabetic animals, it would confirm a reduction of inflammation and bone loss. We believe that the association of metformin with PLGA may confirm the efficacy of metformin in periodontal disease in diabetic rats and enable us to reduce the metformin dosage, while maintaining the same efficacy of inflammation control and bone loss. To answer this question, we aimed to evaluate the effect of metformin hydrochloride-loaded Poly (d,l-Lactide-*co*-glycolide) (PLGA) in a ligature-induced periodontitis model in diabetic rats.

## 2. Results

### 2.1. Characterization of Met-Loaded PLGA (Poly Lactic-co-Glycolic Acid) Nanoparticles

**Figure 1:**
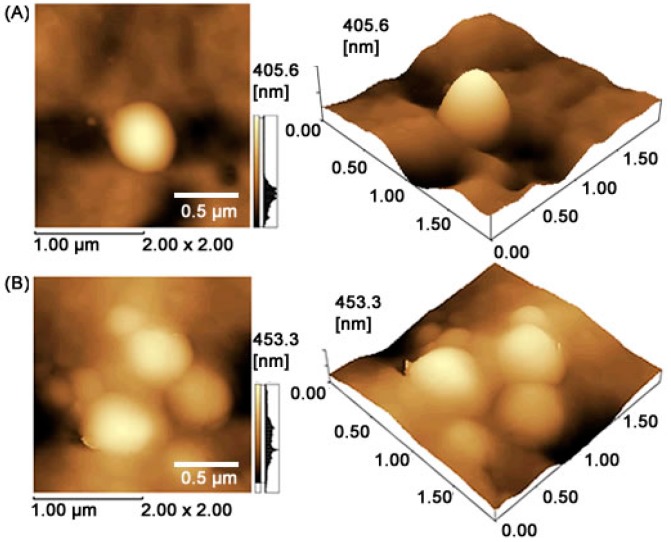
Atomic force microscope (AFM) 2D and 3D images. (**A**) Free drug nanoparticles and (**B**) MET-loaded PLGA (metformin-loaded poly lactic-*co*-glycolic acid) nanoparticles. The topography was observed in water with a frequency shift of 1 Hz and a cantilever oscillation amplitude of 2 µm × 2 µm. The scale indicates the topographic height.

The well-defined spherical morphology and smooth surface of free-drug PLGA nanoparticles and MET-loaded PLGA nanoparticles can be directly observed in an atomic force microscope (AFM) image (Figure 1). Table 1 showed that the mean diameter of MET-loaded PLGA nanoparticles was in the range of 457.1 ± 48.9 nm (*p* < 0.05), with a polydispersity index of 0.285 ± 0.12 (*p* < 0.05), Z potential of 8.16 ± 1.1 mV (*p* < 0.01), and entrapment efficiency (EE) of 66.7 ± 3.73 (Table 1). These results suggest that the addition of MET (metformin) in the core slightly affected the particle sizes (*p* > 0.05). The mean particle size of MET-loaded PLGA nanoparticles was slightly larger than that of pure empty PLGA nanoparticles, indicating the presence of MET in the hydrophilic core of the nanoparticles [16,17].

### 2.2. Glucose Dosing

Induction of diabetes occurred in the control groups (diabetes mellitus (DM), PLGA, and positive control) and also in all treated experimental groups 1 and 2, and diabetes was confirmed for values greater than 300 mL/dL of blood glucose. Glucose levels: Sham group (unbound group), periodontal disease (PD) (bound), DM (diabetic group without ligation), PLGA (diabetic group and with ligation/PLGA DM + PD, diabetic group and with ligation/water), Met 50 (group bound and treated with MET 50 mg/kg), Met 100 (bound and treated group with MET 100 mg/kg), PLGA + 100 mg/kg Met 100 mg/kg + PLGA) and PLGA + 10 mg/kg Met (group bound and treated with MET 10 mg/kg + PLGA). Only treatment with PLGA + 10 mg/kg Met significantly reduced systemic glucose levels in the animals (286.5 ± 109.6 mg/dL, compared with DM (605 + 52.16 mg/dL) and positive control (529.9 + 76.78 mg/dL), *p* < 0.001, Table 2).

### 2.3. Histopathological Analysis

**Figure 2:**
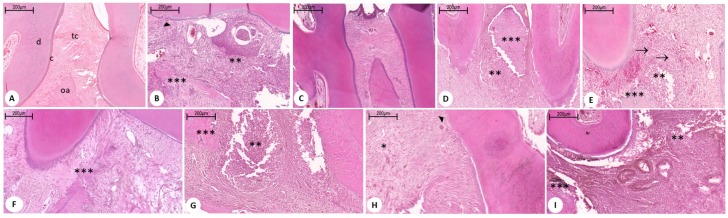
Microscopic analyses. Histopathological aspects of the periodontal control and treated groups. (**A**) Sham Group: Displaying usual aspects; (**B**) PD group without DM; (**C**) DM group without PD: Displaying usual aspects; (**D**) DM group with PD: Positive control; (**E**) Met 50 group; (**F**) Met 100 group: Lower scores; (**G**) PLGA (DM group with PD); (**H**) DM group with PD + PLGA + 10 mg/kg Met group; and (**I**) DM group with PD + PLGA + 100 mg/kg group. Disorganization of connective tissue and intense inflammatory infiltrate (**); alveolar bone destruction (***); (^▸^) destruction of cement; (^→^) Giant multinucleated cell; a: Alveolar bone; c: Cement; d: Dentin; p: Pulp; tc: Connective tissue. * *p* < 0.05, ** *p* < 0.01, *** *p* < 0.00. Hematoxylin and eosin stain (H&E), 200×; PD = periodontal disease; DM = diabetes mellitus.

**Figure 3:**
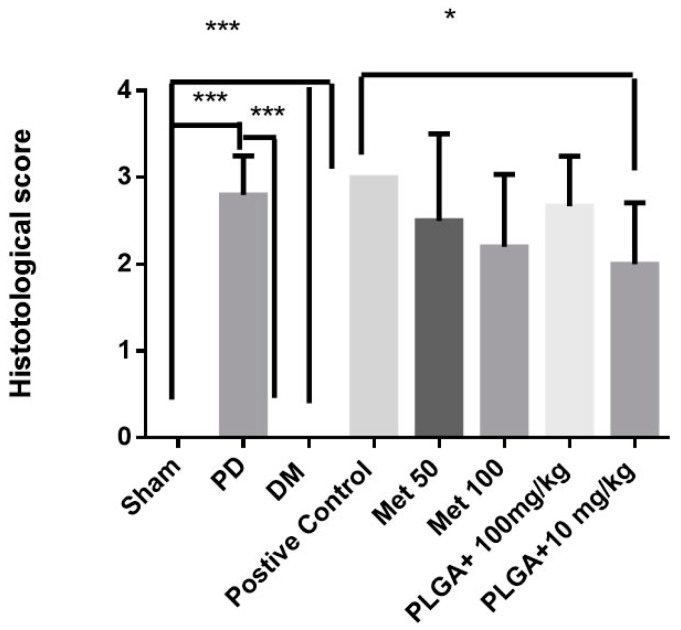
Histopathological score. Sham Group, PD group without DM; DM group without PD; DM group with PD: positive control; Met 50; Met 100; PLGA + 10 mg/kg Met; PLGA + 100 mg/kg. * *p* < 0.05; *** *p* < 0.001. (Kruskal–Wallis test followed by Dunn’s test).

Histopathological data for the Sham and DM control groups showed that infiltration of inflammatory cells was absent or scarce and was restricted to the marginal gingival region, and that the alveolar bone and cement were preserved with scores 0 (0–0) for both groups; the difference was significant when compared to the PD, PLGA control, and positive control groups (*p* < 0.001), Figure 2 and Figure 3. The PD, PLGA control, and positive control groups presented scores of 2.8 (2.5–3.0), 3 (3–3), and 3 (3–3), respectively, with presence of marked infiltration of inflammatory cells in the gingiva and periodontal ligament, marked degradation of the alveolar bone, and partial to severe destruction of dental cement, Figure 2 and Figure 3. The experimental groups Met 50, score: 3 (1.5–3), and PLGA + 100 mg/kg Met, score 3 (2–3) showed a marked inflammatory infiltrate in the gingiva and periodontal ligament, marked degradation of the alveolar process, and partial to severe destruction of the cement, Figure 2 and Figure 3. In turn, the experimental groups Met 100, score: 2 (1.5–3) indicated marked cellular infiltration in the gingiva and periodontal ligament, moderate degradation of the alveolar process, and low cementation, Figure 2 and Figure 3. On the other hand, the PLGA + 10 mg/kg Met, score 2 (1.5–2.5) group indicated moderate inflammatory cellular infiltrate throughout the gingival insertion, light alveolar resorption, and intact cement, with a significant reduction in bone loss when compared to the positive control group (*p* < 0.05), Figure 2 and Figure 3.

### 2.4. Cytokines

**Figure 4:**
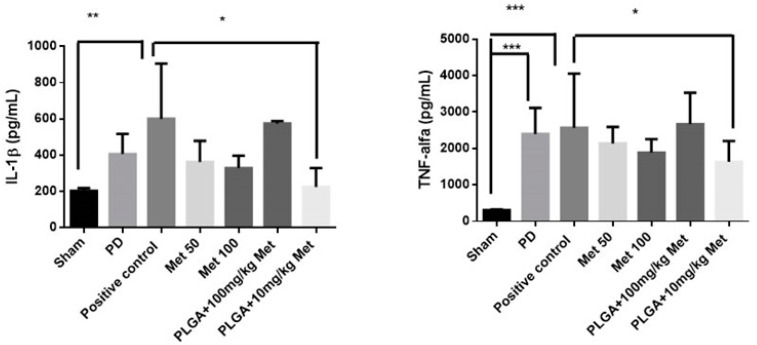
IL-1β and TNF-α levels in Sham, PD; positive control; Met 50; Met 100; PLGA + 10 mg/kg Met; PLGA + 100 mg/kg. * *p* < 0.05, ** *p* < 0.01, *** *p* < 0.001. Analysis of variance/ANOVA test followed by Bonferroni test.

The quantification of inflammatory cytokines showed a significant reduction of IL-1β and TNF-α in the Sham group when compared to the positive control group (*p* < 0.01 and *p* < 0.001, respectively). The quantification of TNF-α showed a significant increase in the PD when compared to the Sham group (*p* < 0.001). The PLGA + 10 mg/kg Met treatment significantly reduced the IL-1β and TNF-α levels when compared to the positive control group (*p* < 0.05), Figure 4.

### 2.5. RT-PCR

**Figure 5:**
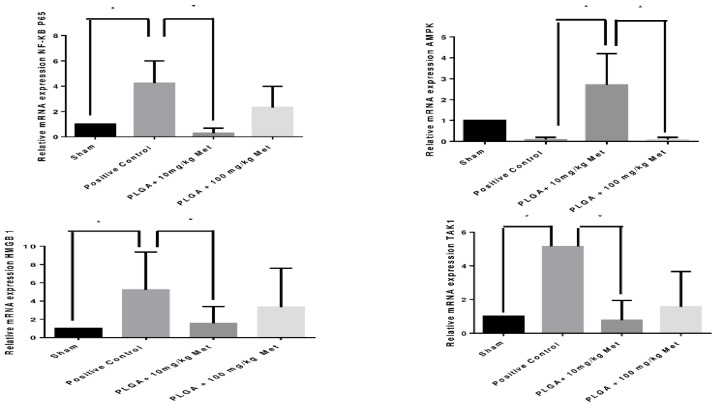
Real-time RT-PCR analysis of gene expression. Met effects on *NF-κB p65*, *AMPK, HMGB1*, and *TAK-1* mRNA expression in rats. The expression of *NF-κB p65* mRNA was decreased in PLGA + 10 mg/kg compared to the positive control group (* *p* < 0.05). The expression of *AMPK* mRNA was increased in PLGA + 10 mg/kg group (* *p* < 0.05) and decreased in positive group and PLGA + 100 mg/kg group. *HMGB1* mRNA levels appeared to be lower in the PLGA + 10 mg/kg group compared to those in the positive control group (* *p* < 0.05,). *TAK-1* appeared to be lower in the PLGA + 10 mg/kg group compared to that in the positive control group (* *p* < 0.05,) (*n* = 5 animals per group; ANOVA test followed by Bonferroni).

Quantification of gene expression for the inflammatory *NF-κB p65* transcription factor showed a significant reduction for the PLGA + 10 mg/kg Met groups (*p* < 0.05) compared to the positive control, Figure 5. Quantification of the gene expression of protein kinase AMP-activated catalytic subunit α 1 (*AMPK*) showed a significant increase in this transcription factor for diabetic animals and periodontal disease treated with PLGA + 10 mg/kg Met when compared to the positive control and PLGA + 100 mg/kg Met (*p* < 0.05) groups, Figure 5. Quantification of *HMGB1* gene expression showed a significant reduction for the PLGA + 10 mg/kg Met groups (*p* < 0.05) when compared to the positive control, Figure 5. The quantification of *TAK1* gene expression showed a significant reduction for the PLGA + 10 mg/kg Met groups (*p* < 0.05) when compared to the positive control, Figure 5.

### 2.6. Radiographic Assessment of Alveolar Bone Loss

**Figure 6:**
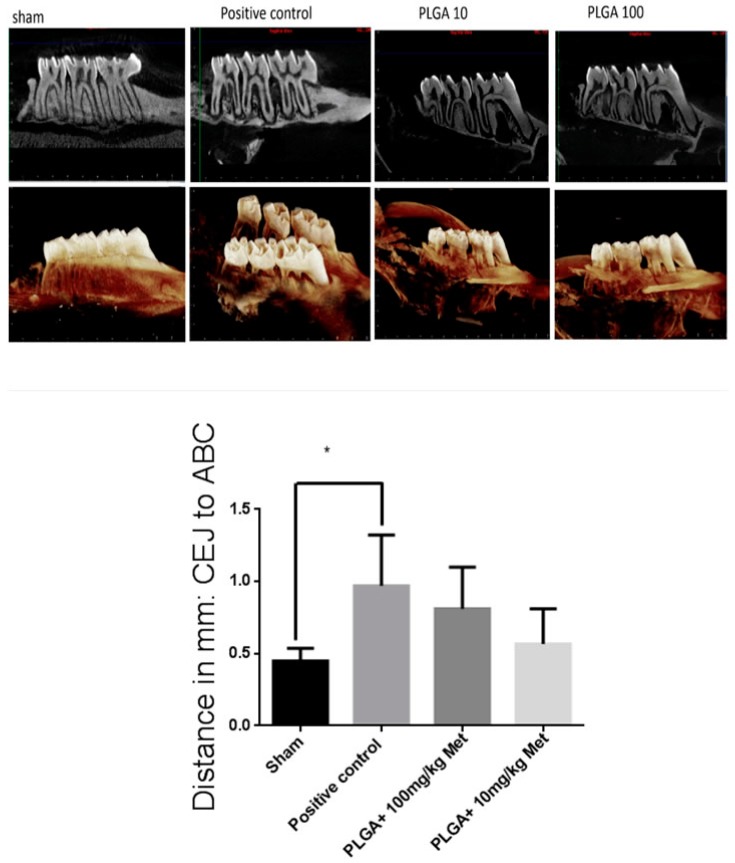
Radiographic evaluation after Met treatment and experimental periodontitis. Representative sagittal 2D and 3D microCT images of no ligature (Sham), ligature and diabetes mellitus (positive control), PLGA + 100 mg/kg Met, and PLGA + 10 mg/kg Met groups. Graph representing linear bone loss in the area of the mesial and distal second molar. Values are expressed as means ± SEM (compared to positive control * *p* < 0.05) (ANOVA test followed by Bonferroni).

Rats with PD + DM (positive control) (0.97 ± 0.35 mm) showed statistically significant more linear bone loss compared to Sham (0.45 ± 0.08 mm), *p* < 0.05. However, bone loss was reduced when comparing positive control (0.97 ± 0.35 mm) to PLGA 10 mg/kg Met (0.48 ± 0.14 mm) treatment. PLGA + 100 mg/kg Met showed bone loss (0.81 ± 0.28 mm) (Figure 6).

### 2.7. Immunohistochemistry

**Figure 7:**
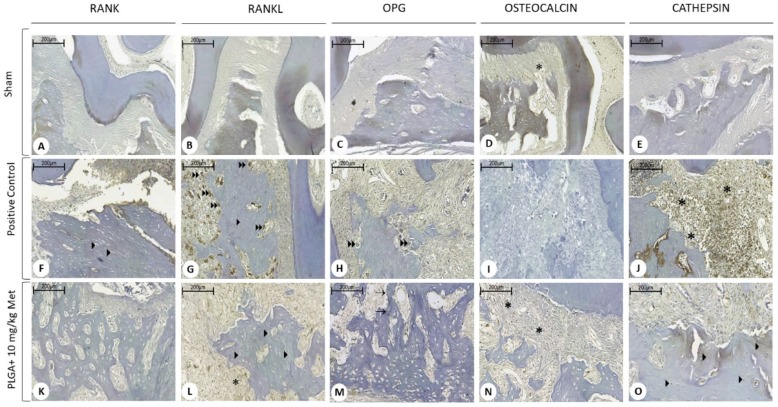
Photomicrographs of periodontal tissue of PD rats treated with PLGA + 10 mg/kg Met, Sham, and positive control groups, showing immunoreactivity to RANK, RANK-L, OPG, cathepsin K, and osteocalcin. Legend: (**A**–**E**) Saline group with discrete immunoblotting for osteocalcin. (**F**–**J**) Positive control group exhibiting discrete immunoblotting for RANK and OPG, intense for RANKL and cathepsin. (**K**–**O**) PLGA + 10 mg/kg Met group demonstrating discrete immunoblotting for RANKL, OPG, osteocalcin, and cathepsin. ^∗^ inflammatory cells; ^▸^ osteocytes; ^
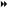
^ osteoclasts; ^→^ osteoblasts. Scale bar: 200×.

**Figure 8:**
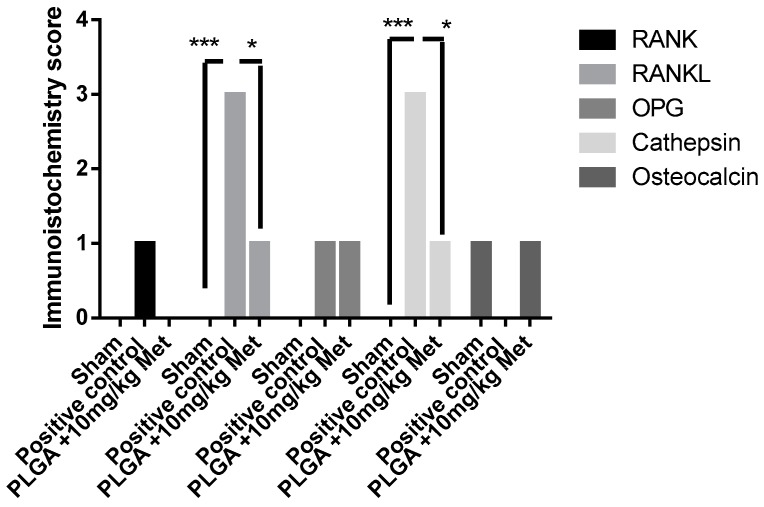
Immunohistochemistry score. Sham, positive control; PLGA + 10 mg/kg Met (* *p* < 0.05). (*** *p* < 0.001) (Kruskal–Wallis test followed by Dunn’s test).

The sham group showed immunostaining absence for RANK, RANKL, OPG, and cathepsin, and low staining for osteocalcin. The positive control group showed intense immunostaining for RANKL and cathepsin, more significantly than the Sham group (*p* < 0.001) and the PLGA + 10 mg/kg Met group (*p* < 0.05). The PLGA + 10 mg/kg Met treatment resulted in low staining of RANKL, OPG, osteocalcin, cathepsin, and osteocalcin (Figure 7 and Figure 8). Immunohistochemical analysis revealed nuclear and cytoplasmic immunoexpression in periodontal connective and bone tissue cells with a diffuse pattern of expression for all proteins in the evaluated specimens, with increased expression in the PLGA + 10 mg/kg Met group compared to positive control. Immunoreactivity to RANK was predominantly observed in osteocytes. RANK-L presented immunoexpression in osteocytes, osteoclasts, and mononuclear inflammatory cells. OPG demonstrated immunostaining in mononuclear inflammatory cells, osteoclasts, and also in osteoblasts present in the PLGA + 10 mg/kg Met group, where an area suggestive of osteoblastic paving was observed. Expression for osteocalcin was present in mononuclear inflammatory cells and cathepsin revealed immunoexpression in mononuclear inflammatory cells and osteocytes.

## 3. Discussion

Polylactic acid contains an asymmetric α-carbon which is typically described as the D or L form in classical stereochemical terms. The enantiomeric forms of the polymer polylactide (PLA) are poly d-lactic acid (PDLA) and poly l-lactic acid (PLLA). PLGA is generally an acronym for poly d,l-lactic-*co*-glycolic acid, where d- and l-lactic acid forms are in equal ratio [7]. PLGA can be processed into almost any shape and size, and can encapsulate molecules of virtually any size. The mechanical strength of PLGA is affected by physical properties, such as molecular weight and polydispersity index [7]. These properties also affect the ability to be formulated as a drug delivery device and may control the device degradation rate and hydrolysis. When copolymerized with PLA, crystalline polylactide (PGA) reduces the crystallinity degree of PLGA and, as a result, increases the hydration and hydrolysis rates. As a rule, higher PGA content leads to quicker degradation rates, with an exception of a 50:50 ratio of PLA/PGA, which exhibits the fastest degradation with higher PGA content leading to an increased degradation interval below 50 [18]. It has a slow and incomplete absorption following oral administration, and repeated applications of high metformin doses are needed for effective treatment, due to its short biological half-life. The development of drug delivery system (e.g., nanoparticles) strategies for metformin might be useful to reduce the doses and dosing frequency [11]. Results of cellular and mitochondrial uptake showed that the metformin-solid lipid nanoparticles (SLNs) were easier to uptake in cells and mitochondria than the pure metformin group [19]. In this study, MET-loaded PLGA nanoparticles with a 50:50 ratio were in the range of 457.1 ± 48.9 nm, with a polydispersity index of 0.285, Z potential of 8.16 ± 1.1 mV, and entrapment efficiency (EE) of 66.7%. These results suggest that the addition of MET in the core slightly affected the particle sizes.

Our data showed that the association of metformin with PLGA was able to reduce glucose levels of 529.9 dL/mL^3^ (positive control group) to 286. 5 dL/mL^3^ (MET 10 + PLGA group), demonstrating that the incorporation of drugs into nanoparticles may improve the drug efficacy [17]. A low polydispersion coefficient guarantees greater control in the size particle and, consequently, in the drug release that the system can supply in biological fluids [7]. Once in the bloodstream, nanoparticles tend to accumulate in places of high blood perfusion, such as organs, tissues affected by inflammation, and tumors [20]. 

It is interesting to highlight that we used the same formulation in two different dilutions (PLGA + 10 mg/kg MET or PLGA + 100 mg/kg Met groups). Some drawbacks of the enhanced permeability and retention (EPR) effect of nanoparticles corroborate these phenomena. When a large amount of hydrophobic nanoparticles is administered in a short volume of medium (as occurs with a dose of PLGA + 100 mg/kg Met), their accumulation in a specific administration site is possible, reducing the surface area and, consequently, the drug diffusion rate [21]. Their drug response is not necessarily dose-dependent and linear, but is dependent on the drug release rate that the polymeric system can supply in a biological medium [22,23,24], and is not necessarily dose-dependent. The lower doses can have the benefit of the EPR effect, as the released drug is able to diffuse more easily. These results indicate that incorporation of the drug into the nanoparticle has systemic benefits, thus favoring glycemia control with a dose reduction of metformin.

PLGA is the most successful and most featured polymer for drug-controlled release systems. The effects of the combination of metformin and PLGA can be observed in both the inflammatory process and in the reduced bone loss in periodontal disease. The histopathological findings show a reduction of the inflammatory infiltrate, little destruction of the periodontal ligament, and absence of dental cement impairment in the group treated with metformin at the dose of 10 mg/kg + PLGA.

This association showed excellent results in periodontal disease in diabetic animals; clinical and histopathological data corroborate the findings that elucidate the main cellular and molecular mechanisms involved in controlling inflammation and bone loss in periodontal disease.

In general, drugs are small enough molecules to cross the endothelium in almost all regions of the body after systemic administration, and can reach both target regions and other healthy regions not affected by any disease, thus causing associated side effects of medication. The use of colloidal nanoparticle systems helps to control these adverse effects and improves therapeutic efficacy. These drugs are encapsulated within nanoparticles of 50–800 nm, which are not able to cross vessel walls of healthy regions of the body (the space between these cells is only 15–30 nm) [25]. Nanoformulation also demonstrates increased anti-inflammatory effects and drug retention at the action site [26]. The present study showed that all groups treated with metformin significantly reduced the inflammatory markers (IL-1β and TNF-α) with a prominence of MET 10 mg/kg + PLGA.

The RANKL, RANK, and OPG system represents the key molecular regulation of bone remodeling [27]. Studies have shown a favorable effect of metformin on bone formation. There are two action mechanisms suggested for the osteogenic effect of metformin, which are increased osteoblast proliferation and decreased osteoclast activity. Studies indicate that the proliferation of Metformin is increased after its absorption by osteoblasts [28]. This drug negatively regulates RANKL production and positively regulates osteoprotegerin (OPG) production from osteoblasts [29]. Thus, there is a decrease in osteoclast activity through this decrease in the RANKL/OPG ratio, aiding in inducing bone formation and inhibiting resorption [30]. In our study, it was found in vivo that metformin, at the dose of 10 mg/kg + PLGA, reduced bone loss with increased osteocalcin immunoblotting and reduced RANKL, indicating an increase in mature osteoblasts and a reduction in the number of osteoclasts.

AMP-activated protein kinase (AMPK) has emerged as a detection mechanism in regulating cellular energy homeostasis and is an essential mediator of the central and peripheral effects of many hormones in glucose metabolism [31]. It is a key molecule in controlling metabolic diseases, such as type 2 diabetes and obesity, and is activated by antidiabetic drugs, such as metformin and thiazolidinediones [32]. Most isoforms of AMPK subunits are expressed in bone cells and bone tissue. It was observed in vitro that metformin (50 µM) significantly increased the expression of osteocalcin, stimulated alkaline phosphatase activity, and increased cell mineralization, yet significantly activated AMPK in a dose- and time-dependent manner [33]. AMPK plays a critical role as a negative feedback regulator of RANKL osteoclast formation promoting action [34]. Araújo et al. (2017) [15] demonstrated that a low dose of metformin reduced bone loss, decreased RANKL, and increased the relative expression of *AMPK* mRNA. Osteocalcin is specifically expressed in osteoblasts, secreted in circulation, and can regulate glucose homeostasis. Metformin stimulates the expression of osteocalcin and the differentiation of osteoblasts via AMPK activation [33]. In our study, it was observed that MET 10 mg/kg + PLGA activated *AMPK* gene expression, which led to stimulated osteocalcin expression and, consequently, osteoblast deposition and bone formation. On the other hand, the increase of AMPK contributed to weak immunolabeling of RANKL and, consequently, of the osteoclast activity.

Lee et al. 2010 [34] demonstrated that AMPK acts via CaMKK and TAK1 activation to serve as a negative feedback regulator of RANKL-induced osteoclast formation. Mizukami et al. [35] also reported that RANKL stimulation facilitates the formation of a complex containing RANK, TRAF6, TAB2, and TAK1, leading to the activation of TAK1. RANKL also acts through TRAF6 to activate TAK1, promoting osteoclastogenesis via NF-κB activation. More interestingly, CaMKK and TAK1 can be activated by RANKL in osteoclast precursors.

HMGB1 acts by stimulating the differentiation of osteoclast precursors in the presence of RANKL, and has similar proinflammatory properties to cytokines once it enters the extracellular space [36]. Thus, in our study, we were able to observe a reduction in HMGB1 at the dose of MET 10 mg/kg, thus showing its relation with inflammation control and in bone loss, since it contributed to a decrease the NFKβ and RANKL levels. 

This study showed that the PLGA + 10 mg/kg Met association had better results, as it managed to control blood glucose levels below what is considered diabetes, and so this nanotechnology product guaranteed rational release of the drug at the inflammation site, thereby controlling inflammation and bone loss in the experimental periodontal disease model.

## 4. Material and Methods

Metformin hydrochloride was purchased from Companhia da Fórmula, d,l-PLGA 50:50 (inherent viscosity of 0.63 dL·g^−1^ at 30 °C) was purchased from Birmingham Polymers Inc. (Birmingham, AL, USA), polyvinyl alcohol (PVA) was purchased from Sigma-Aldrich Co. (St. Louis, MO, USA), and dichloromethane (DCM) from QHEMIS^®^ (Indaiatuba, Brazil). Purified water (1.3 µS·cm^−1^) was prepared from reverse osmosis purification equipment, (OS50 LX Gehaka, São Paulo, Brazil). All other reagents were of analytical grade.

### 4.1. Experimental Design and Preparation of MET-Loaded PLGA Nanoparticles

PLGA nanoparticles for metformin encapsulation were fabricated by adapting the double emulsion solvent diffusion method [37] with some modifications: 50 mg of PLGA was dissolved in 6 mL of dichloromethane (DCM). Metformin (272 mg/mL) was dissolved in an aqueous phase containing 0.1% polyvinyl alcohol. The aqueous phase with the drug (600 µL) was added into 3 mL of organic phase containing PLGA. The mixture was emulsified with a probe-tip sonicator (probe-tip diameter: 1.3 cm, Sonics & Materials Inc., Danbury, CT, USA) operating at 50% amplitude intensity for 1 min. This first mixture was then added into 6 mL of water containing 1.0% of PVA and the mixture was emulsified with a probe-tip sonicator for 1 min. This emulsion was added into 8 mL of water containing 1.0% PVA under magnetic stirring, leading to the formation of a Water/Oil/Water (W/O/W) emulsion with MET-loaded PLGA nanoparticles. The organic solvent was evaporated overnight by magnetic stirring. Free-drug nanoparticles were prepared using the same procedure, but excluding the drug. 

### 4.2. Physicochemical Aspects

The measurements of mean diameter and particle size distribution were assessed by dynamic light scattering in a ZetaPlus device (Brookhaven Instruments Co., New York, NY, USA) equipped with a 90Plus/BI-MAS apparatus at a wavelength of 659 nm, with a scattering angle of 90°. Z potential of the particles was measured by laser Doppler anemometry using the same equipment. All analyses were performed at 25 °C. Experimental values were given as the mean ± SD for the experiments and carried out in triplicate for each sample. The shape and surface of drug-free and MET-loaded nanoparticles were observed using AFM images. The dispersions were freshly diluted with purified water at a ratio of 1:25 (*v*/*v*) and dropped in a cover slip, dried under a desiccator for 24 h and then analyzed in a Shimadzu SPM-9700 AFM (Tokyo, Japan) at room temperature with a cantilever in noncontact mode at 1 Hz scanning. Samples were prepared using one drop of dispersion, which was placed on a washed microscope slide and dried under a desiccator for 24 h, and then analyzed at 25 °C in a cantilever in noncontact mode.

### 4.3. Drug Loading Efficiency

PLGA nanoparticles were used in this experiment to obtain an efficient drug loading corresponding to 6 mg/mL. Metformin hydrochloride-loaded Poly (d,l-Lactide-*co*-glycolide) (PLGA) nanoparticles (NPs) were assessed by an indirect method, in which dispersions were centrifuged at 16,900 RCF (*g*) per 60 min at 4 °C using an ultra-centrifugal filter (Sartorius^®^, Vivaspin 2, Ultra-15 MWCO 10 kDa). The supernatant was removed and diluted in purified water 1:20 (*v*/*v*) and the measurements were carried out in a UV Thermo Fisher Scientific 60S Evolution Spectrophotometer (Waltham, MA, USA), using previously validated UV spectrophotometry at 232 nm. Entrapment efficiency (EE) was calculated using the following equation: EE% = (total drug—drug determined in the supernatant)/total drug × 100.

### 4.4. In Vivo Experimental Study (Periodontal Disease Experimental Model)

The experiments were performed on male Wistar rats (180–220 g) housed under standard conditions (12 h light/dark, 22 ± 0.1 °C), with ad libitum access to food and water. All animal protocols were approved by the Animal Ethics Committee of the Federal University of Rio Grande do Norte (CEUA, Permit No.: 046/2017, 7 November 2017), Brazil. The anesthesia used to induce periodontal disease by intraperitoneal administration was Ketamine 10% (70 mg/kg, Vetnil, São Paulo, Brazil) and 2% xylazine (10 mg/kg, Vet Brands International, São Paulo, Brazil). The animals were euthanized with 80 mg/kg thiopental (Cristália, São Paulo, Brazil). 

### 4.5. Control and Experimental Groups

**Control Groups:**

Twenty animals without diabetes and without periodontal disease (Sham)

Twenty animals with periodontal disease and without diabetes (PD)

Twenty animals with diabetes and without periodontal disease (DM)

Twenty animals with diabetes and with periodontal disease and treatment with vehicle water (Positive Control). This group, called the Positive Control group, was used to compare with all groups, because it has diabetes and periodontal disease (Positive Control group).

Six animals with diabetes and with periodontal disease and treatment with vehicle: PLGA (PLGA Control); this group was used to confirm the same results of the Positive Control group by histopathological analysis. 

We decided to use the Positive Control group as a standard for all other analyses, avoiding excessive use of animals (following the principles of 3Rs).

**Experimental groups 1**: Metformin hydrochloride

Twenty animals with diabetes and with periodontal disease and Metformin 50 mg/kg/day DOSE (Met 50)

Twenty animals with diabetes and periodontal disease and Metformin 100 mg/kg/day DOSE (Met 100)

**Experimental groups 2**: Metformin hydrochloride-loaded Poly (d,l-Lactide-*co*-glycolide) (PLGA) nanoparticles (NPs)

Using the best histopathological results from experimental groups 1, we defined metformin hydrochloride-loaded Poly (d,l-Lactide-*co*-glycolide) (PLGA) nanoparticles (NPs) (with periodontal disease and diabetes). The best anti-inflammatory dose of Experimental groups 1 was used and a lower 10× dose of metformin (best anti-inflammatory dose of Experimental groups 1) was used.

Twenty animals with diabetes and periodontal disease and 100 mg/kg/day of Metformin-loaded PLGA (PLGA + 100 mg/kg Met).

Twenty animals with diabetes and with periodontal disease and 10 mg/kg/day of Metformin-loaded PLGA (PLGA + 10 mg/kg Met).

### 4.6. Diabetes Induction

Diabetes was induced by administration of Streptozotocin/STZ (Sigma-Aldrisch) (40 mg/kg, ip) through the penile vein, dissolved in sodium citrate buffer (0.01 M, pH 4.5) at the concentration of 40 mg/kg body weight under general anesthesia, with 3% isoflurane inhalation. Glucose was measured by a glycosometer (one touch select simple) after one week of STZ administration. Upon reaching plasma glucose stability of ≥300 mg/dL, the animals were considered diabetic and selected for later periodontal disease studies. A puncture was made in the initial portion of the animal’s tail using a sterile needle and the blood was collected on a reagent strip for glucose determination.

### 4.7. Periodontal Disease Induction

After diabetes confirmation, periodontal disease induction was performed by placing a 3.0 nylon wire on the second left molar of male Wistar rats with the animals under i.p. ketamine (80 mg/kg) and xylazine (10 mg/kg) anesthesia. Oral treatments were performed by gavage 30 min prior to periodontal disease induction, which continued until the 10th day. Euthanasia by thiopental (80 mg/kg) was performed on the 11th day. After sacrificing the animals, the gingival and maxillary tissue samples were sent for analysis. 

### 4.8. Histopathological Analysis (Decalcified Tissue)

Histological analyzes were independently performed by two calibrated pathologists. All groups (controls and experimental) were analyzed by histopathology. This step selected the best results that were used for the subsequent analyses. The sectioning was performed in the morphology laboratory of the UFRN, and the slides were analyzed by light microscopy in the department of Morphology. Five jaws were used per group. Alveolar bone specimens were collected, fixed in 10% buffered neutral formol, and demineralized in 5% nitric acid. The samples were then dehydrated, embedded in paraffin and sectioned along the molars in the mesiodistal plane for hematoxylin and eosin. Sections (4 µm) corresponded to the area between the first and second maxillary molars, where ligation was placed for analysis by light microscopy (40× magnification). Influx of inflammatory cells and alveolar bone integrity and cement were analyzed. A score of 0 indicated that infiltration of inflammatory cells was absent or scarce and was restricted to the marginal gingival region, and that the alveolar process and cement were preserved; a score of 1 indicated moderate cell infiltration throughout the gingival insert, minor alveolar resorption, and intact cement; a score of 2 indicated marked cellular infiltration in the gingiva and periodontal ligament, marked degradation of the alveolar process, and partial destruction of the cement; and 3 indicated marked cellular infiltration, complete reabsorption of the alveolar process, and severe destruction of the cement.

### 4.9. Elisa Immunoassay for Detection of IL-1β and TNF-α

Gingival tissues (*n* = 5) of the control (Sham, PD, and positive control) and experimental groups were stored at −70 °C. IL-1β levels (detection range: 62.5–4000 pg/mL; lower detection limit: 12.5 ng/mL of recombinant mouse IL-1β) and TNF-α (detection range: 62.5–4000 pg/mL; lower limit of detection: 50 ng/mL mouse TNF-α recombinant) were determined using commercial ELISA kits (R&D Systems, Minneapolis, MN, USA), as previously described [15]. All samples were measured at 490 nm.

First, microtiter plates were coated overnight at 4 °C with rat antibodies against IL-1β and TNF-α. Plates were then blocked, samples and standards added in several dilutions, in duplicate and incubated at 4 °C for 24 h. The plates were washed three times with buffer and the antibodies were added to the wells (anti-TNF-α or anti-IL-1β, sheep biotinylated polypropylene, diluted 1000 with 1% BSA assay buffer). Plates were incubated at room temperature for 1 h, washed, and 50 µL of avidin-HRP (1:200) µL were added. Then, *O*-phenylenediamine reagent coloring (50 mL) was added 15 min later, and the plates were incubated in the dark at 37 °C for 15–20 min. The absolute quantitative of IL-1β and TNF-α to control (PD and Positive control) and experimental groups was calculated using the comparison with the control group (Sham). The enzymatic reaction was reduced with H_2_SO_4_ and the absorbance measured at 490 nm. Values were expressed in pg/mL.

#### Genetic Marker Rt-PCR Analysis for Periodontal Disease in Diabetic Animals

The control (sham and positive control) and experimental groups (PLGA + 100 mg/kg Met and PLGA + 10 mg/kg Met) were included in the quantification of expression by RT-PCR. Total RNA from the gingival tissues of the treated groups was extracted using the Trizol reagent (Invitrogen, Carlsbad, CA, USA) according to the manufacturer’s guidelines and stored at −70 °C.

RNA concentration was determined from the optical density at a wavelength of 260 nm (using an OD_260_ unit equivalent to 40 µg/mL RNA). Five micrograms of isolated total RNA were reverse transcribed to cDNA in a reaction mixture containing 4 µL 5× reaction buffer, 2 µL dNTP mixture (10 mM), 20 units of RNase inhibitor, 200 units of avian-myeloblastosis virus (AMV) reverse transcriptase, and 0.5 µg oligo (dT) primer (High-Capacity cDNA Reverse Transcription Kit, Foster City, CA, USA) in a total volume of 20 µL. The reaction mixture was incubated at 42 °C for 60 min, and the reaction was terminated by heating at 70 °C for 10 min. The cDNA was stored at −80 °C until further use. Gene expression was evaluated by PCR amplification using primer pairs based on published rat sequences (*GADPH*-*Rattus norvegicus*: Forward primer: AACTTGGCATCGTGGAAGG, Reverse Primer: GTGGATGCAGGGATGATGTTC; AMPK-*Rattus norvegicus* protein kinase, *AMP*-activated, α 2 catalytic subunit (Prkaa2), mRNA: Forward primer: AGCTCGCAGTGGCTTATCAT, Reverse Primer: GGGGCTGTCTGCTATGAGAG; *NF-κB p65*-*Rattus norvegicus* v-rel avian reticuloendotheliosis viral oncogene homolog A (Rela), mRNA Forward primer: 5′-TCTGCTTCCAGGTGACAGTG-3′, Reverse Primer: 5′-ATCTTGAGCTCGGCAGTGTT-3′; *HMGB1-Rattus norvegicus* high mobility group box 1 (*HMGB1*), mRNA: Forward primer: 5′-GAGTACCGCCCAAAAATCAA-3′, Reverse Primer: 5′-TTCATCCTCCTCGTCGTCTT-3′; *TAK-1* Forward primer: 5′-GTCATCCAGCCCTAGTGTCAGATT-3′, Reverse Primer: 5′-TTCTTTGGAGTTTGGGCACG-3′. Transforming Growth Factor β-activated Kinase 1 *TAK-1*-Mus musculus, mRNA: Forward primer: 5′-GTCATCCAGCCCTAGTGTCAGAAT-3′, Reverse Primer: 5′-TTCTTTGGAGTTTGGGCACG-3′.

Quantitative RT-PCR was performed using Power SYBR Green master mix (Applied Biosystems, Waltham, MA, USA), and a Step One Plus thermocycler (Applied Biosystems), according to the manufacturer’s instructions. For the 1× PCR master mix, 2.5 µL of each cDNA was added in a final volume of 20 µL. The PCR conditions were as follows: 95 °C for 5 min, 40 cycles of 30 s at 95 °C, 30 s at 52–60 °C (based on the target), and 60 s at 72 °C. The relative quantitative fold change compared with the control (Sham) was calculated using the comparative *C*_t_ method, where *C*_t_ is the cycle number at which fluorescence first exceeds the threshold. The *C*_t_ values from each sample were obtained by subtracting the values for GADPH *C*_t_ from the target gene *C*_t_ value. The specificity of resulting PCR products was confirmed by melting curves.

### 4.10. Radiographic Microcomputed Tomography (Micro-CT) Measurement of Abl

In this stage, the control groups (Sham and positive control) and experimental groups (PLGA + 100 mg/kg Met and PLGA + 10 mg/kg met) were included. Animals were euthanized at the end of the experiment (10 days after addition of the ligature and first drug treatments); maxillae were dissected and fixed in 10% buffered formalin for 24 h and stored in 70% alcohol. Rat maxillae were scanned using microcomputed tomography (µCT, micro-CT) (Model 1172; SkyScan, Kontich, Belgium) at 20 micrometers resolution. Micro-CT files were converted to Digital Imaging and Communications in Medicine (DICOM) files and imported into Dolphin^®^ software (Toronto, ON, Canada, Version 6.5) for linear bone height analysis. Linear bone height analysis was performed by positioning the second molar cementoenamel junctions (CEJ) parallel to each other in the coronal plane. In the axial plane, the middle of the crown was identified and linear bone distances were recorded on the mesial aspect of the second molar on the sagittal image. Additional measurements were taken 0.3 mm palatal from the middle of the crown, again recording the mesial aspect of the second molar on the sagittal image. The linear measurements were recorded from the CEJ to the alveolar crest (AC). Each second molar received a total of two measurements, and these values were averaged for each group. The images were analyzed using CTAn (V.1.16 Bruker, Billerica, MA, USA). A 40-slice volume set at a threshold of 75 in the bifurcation area of the second molar was used as a region of interest for analysis (*n* ≥ 3/group for all µCT analyses).

### 4.11. Immunohistochemistry

Only the controls (Sham and positive control) and the experimental group (PLGA + 10 mg/kg met) were included in this step. Fine sections of periodontal tissue (4 µm) (3 mandibles per group) were produced using a microtome and transferred onto gelatin coated slides. Each section was deparaffinized and rehydrated. Gingival and periodontal tissues were washed with 0.3% Triton X-100 in phosphate buffer, then extinguished with peroxidase (3% hydrogen peroxide) and incubated with the following primary antibodies (Santa Cruz Biotechnology, Interprise, Brazil) overnight at 4 °C: RANKL, 1400; OPG, 1400; cathepsin K, 1400; and osteocalcin, 1400, which were washed with phosphate buffer and incubated with streptavidin-HRP-conjugated secondary antibodies (Biocare physicians, Concord, CA, USA) for 30 min, and immunoreactivity for RANK, RANK-L, OPG, cathepsin K, and osteocalcin were visualized using a colorimetric detection kit following the manufacturer’s instructions (TrekAvidin-HRP Label, Biocare Medical, Pacheco, CA, USA; TrekAvidin-HRP Kit, Dako, Carpinteria, CA, USA).

### 4.12. Statistical Analysis

Using nanoparticle characterization, pairwise comparisons of the analytical data were performed using the Student’s *t*-test. One-way analysis of variance (ANOVA) was applied for multiple comparisons, followed by Tukey’s post hoc test. *p* < 0.05 was considered statistically significant. Data for the in vivo experiments were analyzed using descriptive and analytical statistics. Parametric tests, such as ANOVA, followed by Bonferroni’s post-test and nonparametric Kruskal–Wallis test were used. A significance level of 5% was considered.

## Figures and Tables

**Table 1:** Loading efficiency of metformin-loaded PLGA (metformin-loaded poly lactic-*co*-glycolic acid) nanoparticles by double emulsification.

Formulation	Particle Size (nm)	PDI	Zeta Potential (mV)	EE (%)
NP Empty	406.3 ± 14.5	0.187 ± 0.01	−1.51 ± 3.2	−
NP + MET	457.1 ± 48.9 *	0.285 ± 0.12 *	8.16 ± 1.1 **	66.7 ± 3.73

Notes: NP, nanoparticles; PDI, polydispersity index; MET, metformin; EE, encapsulation efficiency. The results are expressed as mean ± SD (*n* = 3); * *p* < 0.05; ** *p* < 0.01.

**Table 2:** Glucose of animals/group, Natal, RN, Brazil, 2018.

Groups	Glucose mg/dL (Mean ± Standard Deviation)
Sham	115.7 ± 18.86 ^a^ ***^, b^ ***
PD	176.7 ± 90.4 ^a^ ***^, b^ ***
DM	605 ± 52.16
Positive control/(PD + DM)	529.9 ± 76.78
Met 50	523.2 ± 31.74
Met 100	522.0 ± 78.32
PLGA	512 ± 56.02
PLGA + 10 mg/kg Met	286.5 ± 109.6 ^a^ ***^, b^ ***
PLGA + 100 mg/kg Met	440 ± 59.9

^a^ = Difference among groups and DM, ^b^ = difference among groups and positive control/PD + DM (Periodontal disease + Diabetes mellitus), *** *p* < 0.001.

## Abbreviations

PLGA	Poly (d,l-Lactide-*co*-glycolide)
NPs	Nanoparticles
Met	Metformin
AFM	Atomic force microscopy
EPR	Enhanced permeability and retention effect
μCT	Microcomputed tomography

## References

[1] Freichels H., Danhier F., Préat V., Lecomte P., Jérôme C. (2011). Fluorescent labeling of degradable poly(lactide-*co*-glycolide) for cellular nanoparticles tracking in living cells. Int. J. Artif. Organs.

[2] Jia L. (2005). Nanoparticle formulation increases oral bioavailability of poorly soluble drugs: Approaches experimental evidences and theory. Curr. Nanosci..

[3] Lai F., Schlich M., Pireddu R., Corrias F., Fadda A.M., Sinico C. (2015). Production of nanosuspensions as a tool to improve drug bioavailability: Focus on topical delivery. Curr. Pharm. Des..

[4] Sharma A., Madhunapantula S.V., Robertson G.P. (2012). Toxicological considerations when creating nanoparticle based drugs and drug delivery systems?. Expert Opin. Drug Metab. Toxicol..

[5] Liu K.C., Yeo Y. (2014). Extracellular stability of nanoparticulate drug carriers. Arch. Pharm. Res..

[6] Zhou Y., Zhang L., Zhao W., Wu Y., Zhu C., Yang Y. (2013). Nanoparticle-mediated delivery of TGF-β1 miRNA plasmid for preventing flexor tendon adhesion formation. Biomaterials.

[7] Makadia H.K., Siegel S.J. (2011). Poly Lactic-*co*-Glycolic Acid (PLGA) as biodegradable controlled drug delivery carrier. Polymers.

[8] Kobayashi M., Yamazaki K., Hirao K., Oishi M., Kanatsuka A., Yamauchi M., Takagi H., Kawai K., Japan diabetes clinical data management study group (2006). The status of diabetes control and antidiabetic drug therapy in Japan—A cross-sectional survey of 17,000 patients with diabetes mellitus (JDDM 1). Diabetes Res. Clin. Pract..

[9] Gelperina S., Kisich K., Iseman M.D., Heifets L. (2005). The potential advantages of nanoparticle drug delivery systems in chemotherapy of tuberculosis. Am. J. Respir. Crit. Care Med..

[10] Bucher S., Bauduceau B., Benattar-Zibi L., Bertin P., Berrut G., Corruble E., Danchin N., Delespierre T., Derumeaux G., Doucet J. (2015). Primary care management of non-institutionalized elderly diabetic patients: The S.AGES cohort—Baseline data. Prim. Care Diabetes.

[11] Cetin M., Sahin S. (2016). Microparticulate and nanoparticulate drug delivery systems for metformin hydrochloride. Drug Deliv..

[12] Andrews M., Soto N., Arredondo M. (2012). Effect of metformin on the expression of tumor necrosis factor-α, Toll like receptors 2/4 and C reactive protein in obese type-2 diabetic patients. Rev. Med. Chile.

[13] Koh S.J., Kim J.M., Kim I.K., Ko S.H., Kim J.S. (2014). Anti-inflammatory mechanism of metformin and its effects in intestinal inflammation and colitis-associated colon cancer. J. Gastroenterol. Hepatol..

[14] Tanaka H., Nakai K., Murakami F., Morita T., Yamazaki Y., Matsuike R., Shibata C., Nagasaki M., Kanda M., Tanabe N. (2017). Ligature-induced periodontitis increased insulin resistance and triglyceride levels in wistar rats. J. Hard. Tissue Biol..

[15] De Araújo A.A., Pereira AD SB F., de Medeiros CA C.X., de Castro Brito G.A., de Carvalho Leitão R.F., de Souza Araújo L., Guedes P.M.M., Hiyari S., Pirih F.Q., de Araújo Júnior R.F. (2017). Effects of metformin on inflammation, oxidative stress, and bone loss in a rat model of periodontitis. PLoS ONE.

[16] Martinez N.Y., Andrade P.F., Duran N., Cavalitto S. (2017). Development of double emulsion nanoparticles for the encapsulation of bovine serum albumin. Colloids Surf. B Biointerfaces.

[17] Iqbal M., Zafar N., Fessi H., Elaissari A. (2015). Double emulsion solvent evaporation techniques used for drug encapsulation. Int. J. Pharm..

[18] Miller R.A., Brady J.M., Cutright D.E. (1977). Degradation rates of oral resorbable implants (polylactates and polyglycolates): Rate modification with changes in PLA/PGA copolymer ratios. J. Biomed. Mater. Res..

[19] Xu Q., Zhu T., Yi C., Shen Q. (2016). Characterization and evaluation of metformin-loaded solid lipid nanoparticles for celluar and mitochondrial uptake. Drug Dev. Ind. Pharm..

[20] Kamaly N., Xiao Z., Valencia P.M., Radovic-Moreno A.F., Farokhzad O.C. (2012). Targeted polymeric therapeutic nanoparticles: Design, development and clinical translation. Chem. Soc. Rev..

[21] Danhier F. (2016). To exploit the tumor microenvironment: Since the EPR effect fails in the clinic, what is the future of nanomedicine?. J. Control..

[22] Kohane D.S., Langer R. (2008). Polymeric biomaterials in tissue engineering. Pediatr. Res..

[23] Goldberg M., Langer R., Jia X. (2007). Nanostructured materials for applications in drug delivery and tissue engineering. J. Biomater. Sci. Polym. Ed..

[24] Formariz T.P., Urban M.C.C., Silva Júnior A.A.D., Gremião M.P.D., Oliveira A.G.D. (2005). Microemulsões e fases líquidas cristalinas como sistemas de liberação de fármacos. Rev. Bras. Ciênc. Farm..

[25] Vieira D.B., Gamarra L.F. (2016). Advances in the use of nanocarriers for cancer diagnosis and treatment. Einstein.

[26] Leonard F., Ali H., Collnot E.M., Crielaard B.J., Lammers T., Storm G., Lehr C.M. (2012). Screening of budesonide nanoformulations for treatment of inflammatory bowel disease in an inflamed 3D cell-culture model. ALTEX.

[27] Walsh M.C., Choi Y. (2014). Biology of the RANKL-RANK-OPG system in immunity, bone, and beyond. Front. Immunol..

[28] Bak E.J., Park H.G., Kim M., Kim S.W., Kim S., Choi S.H., Cha J.H., Yoo Y.J. (2010). The effect of metformin on alveolar bone in ligature-induced periodontitis in rats: A pilot study. J. Periodontol..

[29] Liu L., Zhang C., Hu Y., Peng B. (2012). Protective effect of metformin on periapical lesions in rats by decreasing the ratio of receptor activator of nuclear factor κB ligand/osteoprotegerin. J. Endod..

[30] Najeeb S., Zafar M.S., Khurshid Z., Zohaib S., Madathil S.A., Mali M., Almas K. (2018). Efficacy of metformin in the management of periodontitis: A systematic review and meta-analysis. Saudi Pharm. J..

[31] Jeyabalan J., Shah M., Viollet B., Chenu C. (2012). AMP-activated protein kinase pathway and bone metabolism. J. Endocrinol..

[32] LeBrasseur N.K., Kelly M., Tsao T.S., Farmer S.R., Saha A.K., Ruderman N.B., Tomas E. (2006). Thiazolidinediones can rapidly activate AMP-activated protein kinase in mammalian tissues. Am. J. Physiol. Endocrinol. Metab..

[33] Kanazawa I., Yamaguchi T., Yano S., Yamauchi M., Sugimoto T. (2008). Metformin enhances the differentiation and mineralization of osteoblastic MC3T3-E1 cells via AMP kinase activation as well as eNOS and BMP-2 expression. Biochem. Biophys. Res. Commun..

[34] Lee Y.-S., Kim Y.-S., Lee S.-Y., Kim G.H., Kim B.J., Lee S.H., Lee K.U., Kim G.S., Kim S.W., Koh J.M. (2010). AMP kinase acts as a negative regulator of RANKL in the differentiation of osteoclasts. Bone.

[35] Mizukami J., Takaesu G., Akatsuka H., Sakurai H., Ninomiya-Tsuji J., Matsumoto K., Sakurai N. (2002). Receptor activator of NF-κB ligand (RANKL) activates TAK1 mitogen-activated protein kinase kinase kinase through a signaling complex containing RANK, TAB2, and TRAF6. Mol. Cell. Biol..

[36] Zhou Z., Han J.-Y., Xi C.-X., Xie J.X., Feng X., Wang C.Y., Mei L., Xiong W.C. (2008). HMGB1 regulates RANKL-induced osteoclastogenesis in a manner dependent on RAGE. J. Bone Miner. Res..

[37] Palamoor M., Jablonski M.M. (2014). Comparative study on diffusion and evaporation emulsion methods used to load hydrophilic drugs in poly(ortho ester) nanoparticle emulsions. Powder Technol..

